# Antimicrobial Resistance Patterns of ESBL-Producing *Escherichia coli* in Dogs from Thailand: Evaluation of Algal Extracts as Novel Antimicrobial Agents

**DOI:** 10.3390/antibiotics14040377

**Published:** 2025-04-03

**Authors:** Khomson Satchasataporn, Duangdaow Khunbutsri, Peechanika Chopjitt, Samak Sutjarit, Wanida Pan-utai, Nattakan Meekhanon

**Affiliations:** 1 Department of Veterinary Nursing, Faculty of Veterinary Technology, Kasetsart University, Bangkok 10900, Thailand; cvtkss@ku.ac.th (K.S.); cvtsms@ku.ac.th (S.S.); 2 Veterinary Diagnostic Laboratory, Faculty of Veterinary Medicine, Khon Kaen University, Khon Kaen 40002, Thailand; duankh@kku.ac.th; 3 Faculty of Public Health, Kasetsart University, Chalermphrakiat Sakon Nakhon Province Campus, Sakon Nakhon 47000, Thailand; peechanika.c@ku.th; 4 Department of Applied Microbiology, Institute of Food Research and Product Development, Kasetsart University, Bangkok 10900, Thailand

**Keywords:** algal extract, dog, *Escherichia coli*, extended-spectrum beta-lactamase, multidrug-resistant

## Abstract

**Background/Objectives:** Multidrug-resistant (MDR) bacteria, including extended-spectrum beta-lactamase (ESBL)-producing *Escherichia coli*, in companion animals pose a growing public health concern due to the close interactions between pets and humans. This study aimed to investigate antimicrobial resistance patterns and the prevalence of ESBL-producing *E. coli* isolated from healthy dogs in Thailand, as well as the potential of algal extracts obtained through ethanol extraction and enzymatic hydrolysis as alternative antimicrobial agents against these drug-resistant organisms. **Methods:** Antimicrobial susceptibility testing was performed on 43 *E. coli* isolates from healthy dogs. ESBL production was confirmed using standard phenotypic methods, and resistance genes were detected by PCR. The algal extracts were tested for antibacterial activity against MDR isolates. **Results:** Among the 43 *E. coli* isolates, 67.44% were classified as MDR, with high resistance rates observed for ampicillin (79.07%), tetracycline (65.12%), and ciprofloxacin (62.79%), highlighting significant antimicrobial resistance concerns. Of the MDR isolates, 31.03% (9/29) were confirmed as ESBL producers. Gene analysis revealed *bla*_TEM_ as the most prevalent gene (53.49%), followed by *bla*_CTX-M_ (9.30%), while *bla*_SHV_ was detected in a single isolate resistant only to ampicillin and was absent in all MDR strains. Ethanol extracts of *Haematococcus pluvialis* and *Caulerpa lentillifera* demonstrated inhibitory effects against MDR *E. coli*. **Conclusions:** MDR and ESBL-producing *E. coli* are prevalent in healthy dogs, posing a potential public health risk. Algal extracts from *H. pluvialis* and *C. lentillifera* show promise as alternative antimicrobials. Further research is necessary to optimize their efficacy and investigate their in vivo applications, including clinical and environmental settings.

## 1. Introduction

The excessive and inappropriate use of antibiotics has significantly contributed to the accelerated emergence of multidrug-resistant (MDR) bacterial strains, leading to major challenges for healthcare systems globally [1]. This rapid increase in MDR bacteria has raised serious concerns about the use of antibiotics, as emphasized by the World Health Organization (WHO) [2]. Among these, *Enterobacterales* producing extended-spectrum beta-lactamase (ESBL) enzymes are of particular concern, as they make a broad spectrum of beta-lactam antibiotics ineffective. *Escherichia coli*, a common Gram-negative bacterium found in the intestines of warm-blooded animals, is particularly notable for its role in both commensal and pathogenic states. Within the intestinal environment, *E. coli* is exposed to various antimicrobial agents, creating high selective pressure for antibiotic resistance. Moreover, *E. coli* can contaminate the environment, posing risks to public health. Although *E. coli* is typically a commensal organism, it can cause a variety of infections, including diarrhea, pneumonia, meningitis, surgical site infections, and extraintestinal infections [3]. *E. coli* has been identified as a major contributor to the global disease burden, particularly among Gram-negative bacterial infections. In 2019, *E. coli* infections accounted for an estimated 28.5 million Disability-Adjusted Life Years (DALYs) across all age groups, with 10.6 million DALYs specifically affecting children under five years old. Among the top 20 most burdensome pathogens globally, *E. coli* ranked prominently and was among the top 10 pathogens contributing to disease burden in children younger than five years. Additionally, *E. coli* was one of the top five pathogens based on DALY burden, representing 9.8% of cases in 2020. Infections caused by *E. coli*, along with other Gram-negative bacteria, were estimated to collectively account for 114 million DALYs worldwide [4]. The emergence of antibiotic-resistant strains, especially those producing ESBL, complicates treatment and increases the risk of transmission between animals and humans.

The population of companion animals, including dogs, in Thailand has increased over the last few decades, leading to an expansion in both the quality and quantity of pet healthcare services, particularly in metropolitan areas like Bangkok. The frequent use of antibiotics in veterinary medicine has contributed to the development of resistant bacterial strains in these animals. This resistance not only impacts animal health but also poses a risk to public health, as resistant bacteria can spread through direct contact, environmental contamination, and the food chain. Dogs and cats, in particular, act as reservoirs for antimicrobial-resistant bacteria, increasing the risk of zoonotic transmission due to their frequent close interactions with humans and other animals [5]. As traditional antibiotics become less effective, there is a pressing need to explore alternative strategies to address these resistant pathogens. In this context, natural bioactive agents are considered promising candidates for their potential antimicrobial properties.

Algae have long been known for their use as food and medicine. Additionally, their secondary metabolites, including phlorotannins, fatty acids, polysaccharides, peptides, terpenes, polyacetylenes, sterols, indole alkaloids, aromatic organic acids, shikimic acid, polyketides, hydroquinone, alcohols, aldehydes, ketones, and halogenated furanones, alkanes, and alkenes, have demonstrated antibacterial activity [6]. This suggests their potential to be developed as safe alternative antibacterial agents against MDR bacteria. However, research on the potential of algae to produce bioactive compounds that inhibit MDR bacteria remains limited.

This study aims to comprehensively analyze antibiotic resistance and ESBL-producing *E. coli* isolated from healthy dogs and to explore the potential of algae extracts as a defense against these drug-resistant organisms.

## 2. Results

### 2.1. E. coli Detection and Antimicrobial Susceptibility Testing

Of the 53 rectal swab samples, 43 isolates were identified as *E. coli* based on their Gram staining morphology (Gram-negative rods), their growth characteristics on MacConkey agar (rose-pink, dotted colonies) and EMB agar (metallic sheen), and their positive results for indole and methyl red tests, along with negative results for Voges–Proskauer and citrate utilization tests. All isolates tested positive for PCR targeting *uidA*, confirming their identification as *E. coli*.

The agar disk diffusion results demonstrated a high prevalence of multidrug resistance among the bacterial isolates, with 67.44% (29/43) exhibiting resistance to multiple commonly used antibiotics (Table 1). Among the antibiotics tested, meropenem demonstrated the highest susceptibility rate (81.40%), followed by cefepime and amikacin, both at 72.09%. In contrast, ampicillin, tetracycline, and ciprofloxacin showed the highest resistance rates at 79.07%, 65.12%, and 62.79%, respectively. Additionally, 60.47% of isolates were resistant to trimethoprim–sulfamethoxazole, indicating the limited effectiveness of this agent. For the cephalosporin group, resistance rates to third-generation agents, such as cefotaxime (32.56%) and ceftriaxone (32.56%), were nearly double those of cefepime (18.60%), a fourth-generation cephalosporin. Among aminoglycosides, gentamicin demonstrated a higher resistance rate (37.21%) compared to amikacin (16.28%). Meropenem exhibited the lowest resistance rate at 2.33%, while amoxicillin–clavulanate, a β-lactam combination agent, showed a moderate resistance rate of 37.21%.

As shown in Table 2, the multiple antibiotic resistance (MAR) index values ranged from 0 to 0.91, indicating varying degrees of multidrug resistance among the isolates. A MAR index greater than 0.2 is considered a threshold for high-risk contamination sources, as it suggests exposure to environments with frequent or extensive antibiotic use [7]. Based on this criterion, 29 out of 43 isolates (67.44%) were classified as high-risk. Only four isolates (En19, En27, En41, and En51) were susceptible to all tested antimicrobial agents. In contrast, isolate En17 had the highest MAR index of 0.91, followed by isolates En2, En8, En16, En24, and En33, each with a MAR index of 0.82.

### 2.2. ESBL-Producing E. coli

**Figure 1:**
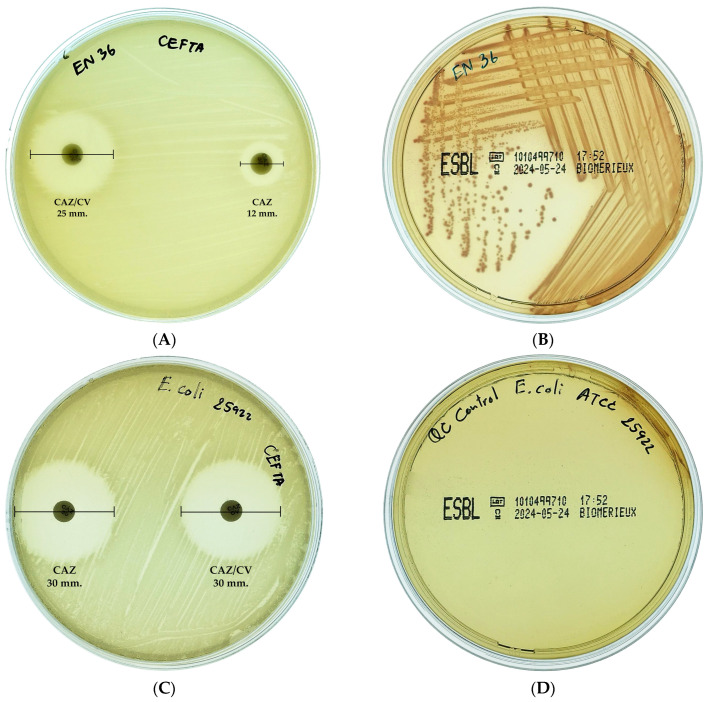
Interpretation of ESBL-producing *E. coli* is illustrated as follows: (**A**) ESBL production was identified by a ≥5 mm increase in zone diameter with clavulanate combination disks. (**B**) Pink to burgundy colonies on CHROMID^®^ ESBL agar confirmed ESBL production. (**C**) In contrast, *E. coli* ATCC 25922 (negative control) was confirmed as non-ESBL-producing, showing a ≤5 mm difference in zone diameters. (**D**) The absence of growth in *E. coli* ATCC 25922 confirmed the absence of ESBL production.

The double-disk synergy test was used to phenotypically screen 29 MDR *E. coli* isolates for ESBL production. Isolates were considered ESBL producers if they exhibited a ≥5 mm increase in zone diameter around the cephalosporin–clavulanate combination disk compared to the cephalosporin disk alone. To further confirm ESBL production, isolates were also cultured on CHROMID^®^ ESBL agar (bioMérieux, Marcy l’Étoile, France), where the development of pink to burgundy colonies indicated a positive result. Among the tested isolates, nine (31.03%) were confirmed as ESBL-producing *E. coli*. A representative interpretation of the test results is illustrated in Figure 1.

### 2.3. Detection of Beta-Lactamase Genes

The detection of beta-lactamase genes in the *E. coli* isolates showed a varied distribution of specific gene types, as presented in Table 2. The *bla*_TEM_ gene was the most prevalent, detected in 53.49% (23/43) of isolates, including some non-MDR strains (En20, En23, En28, En37, and En41). The *bla*_CTX-M_ gene was found in 9.3% (4/43) of isolates, including En8, En29, En32, and En36. Notably, the *bla*_SHV_ gene was detected in only one isolate, which exhibited resistance solely to ampicillin and was absent in all MDR strains. Interestingly, 24.14% (7/29) of MDR strains lacked any detectable beta-lactamase genes, while 42% (6/14) of non-MDR strains harbored these genes.

### 2.4. Effect of Algal Extract on Inhibition of Multidrug-Resistant E. coli Isolates

**Figure 2:**
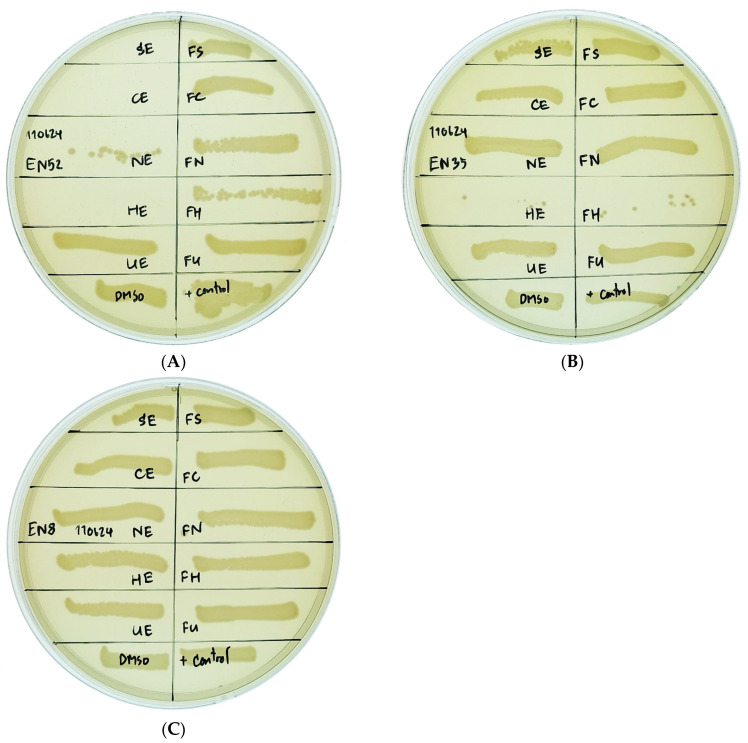
An example of the interpretation of algal extracts on inhibiting multidrug-resistant (MDR) *E. coli* isolates is presented as follows: (**A**) The effects of ethanol extracts from *Haematococcus pluvialis* (HE) and *Caulerpa lentillifera* (CE) demonstrated significant inhibition of growth in the *E. coli* EN52 MDR isolate. (**B**) In contrast, the ethanol extracts of *Haematococcus pluvialis* (HE) and enzymatic hydrolysis of *Haematococcus pluvialis* (FH) resulted in only a slight inhibition of growth in the *E. coli* EN35 MDR isolate. (**C**) Furthermore, the algae extracts showed no inhibitory effect on the *E. coli* EN8 MDR isolate.

The antimicrobial activity of algal extracts against MDR *E. coli* isolates was assessed. Effective growth inhibition of MDR *E. coli* isolates EN16, EN29, and EN52 was observed with the ethanol extract of *Haematococcus pluvialis* (HE), while the ethanol extract of *Caulerpa lentillifera* (CE) effectively inhibited EN52, as indicated by the absence of microorganism growth on the culture medium. In contrast, HE, along with the ethanol extracts of *Arthrospira platensis* (SE) and *Nannochloropsis oculata* (NE), as well as the enzymatically hydrolyzed extracts of *Haematococcus pluvialis* (FH) and *Caulerpa lentillifera* (FC) exhibited only slight inhibitory effects, as evidenced by partial growth of microorganisms on the culture medium. Moreover, the ethanol extract of *Ulva rigida* (UE) and the enzymatically hydrolyzed extracts of *Arthrospira platensis* (FS), *Nannochloropsis oculata* (FN), and *Ulva rigida* (FU) showed no inhibitory activity against MDR *E. coli* isolates, as demonstrated by full microorganism growth on the culture medium (Table 3). An illustrative example of the inhibitory effects of algal extracts on MDR *E. coli* isolates is presented in Figure 2.

## 3. Discussion

The presence of MDR bacteria in pets is a growing concern due to the close relationships shared between pets and humans. MDR bacteria, including ESBL-producing *E. coli*, can be transmitted from pets to humans through both direct and indirect contact [8]. The high prevalence of these resistant strains in healthy dogs indicates that pets may act as reservoirs for MDR bacteria and antimicrobial resistance genes [8], posing a significant risk to human health through direct interaction and environmental contamination [9]. Among the intestinal flora, *E. coli* serves as an important indicator of antimicrobial resistance [10]. Our findings revealed a high prevalence of MDR *E. coli* in healthy dogs, with a rate of 67.44%. While this aligns with global trends indicating an increasing incidence of MDR bacteria in companion animals and high resistance to commonly used antibiotics [11], the percentage of MDR isolates in our study was notably higher than those reported in other countries. For instance, China reported 62.42% MDR isolates in pet dogs in 2021 [12]. South Korea reported 34.90% in *E. coli* isolated from healthy dogs [13], and the United Kingdom reported 30% in isolates from healthy Labrador retrievers [14]. Interestingly, a previous study conducted in other provinces of Thailand reported a lower prevalence of MDR *E. coli* in healthy dogs (34.92%) [15]. The higher prevalence observed in the present study may reflect more extensive antimicrobial use in pets within this specific region. However, due to the small sample size in our study, the findings should be interpreted with caution, as this limitation may introduce bias. Further studies with larger sample sizes across multiple regions are needed to confirm these observations.

The MAR index provides a cost-effective and rapid method for tracking potential sources of antibiotic-resistant bacteria, including *E. coli* and *Salmonella* spp. [7]. A MAR index exceeding 0.2 indicates that the bacterial isolate likely originated from a source with extensive or frequent antibiotic use [16]. In this study, 29 out of 43 *E. coli* isolates (67.44%) from healthy dogs exhibited MAR indices greater than 0.2, indicating a serious public health concern regarding antimicrobial resistance in veterinary settings. This percentage is higher than the 36.89% reported in a previous study in Thailand, which assessed antimicrobial-resistant *E. coli* in pets, veterinary staff, and pet owners [15]. That study found the highest MAR index values in veterinarians, followed by cats. However, our findings are more comparable to a study conducted in China in 2021, where 62.42% of *E. coli* isolates from pet dogs had a MAR index ≥ 0.2 [12]. Due to the close contact between dogs and their owners and family members, there is a potential risk of MDR *E. coli* transmission through the environment. This poses a particular threat to young children, who may be exposed to MDR strains via contaminated food or surfaces, and to elderly individuals, who are more susceptible to infections. These findings emphasize the need for antimicrobial stewardship in veterinary medicine to minimize the emergence and spread of resistant bacterial strains.

Our findings revealed a concerning level of antimicrobial resistance among the *E. coli* isolates from healthy dogs, with high resistance rates observed for ampicillin (79.07%), tetracycline (65.12%), and ciprofloxacin (62.79%). Trimethoprim–sulfamethoxazole also demonstrated notable resistance (60.47%). Our findings are consistent with some previous studies while also highlighting notable differences in resistance patterns. A recent study conducted in five provinces across Thailand reported high resistance rates to tetracycline (42.33%), ampicillin (37.57%), and doxycycline (29.63%), aligning with our findings for ampicillin and tetracycline [15]. Similarly, a study in China also reported high ampicillin resistance in *E. coli* isolates from healthy dogs [12]. However, a study from the United Arab Emirates (UAE) reported markedly higher resistance rates to ampicillin (97.4%), ceftriaxone (94.81%), and cefotaxime (90.91%), along with notable resistance to trimethoprim–sulfamethoxazole (79.22%), tetracycline (58.44%), and ciprofloxacin (57.14%) [17]. In contrast, a study conducted in Korea during 2020–2022 reported cephalexin as the antibiotic with the highest resistance rate (74.40%), followed by ampicillin (42.40%) and tetracycline (25.60%) [13]. These variations in resistance patterns across different studies can be attributed to several factors. Geographical location plays a crucial role, as the level and type of antimicrobial drug usage can vary significantly between regions. The source of samples (e.g., healthy vs. diseased animals), animal species, and the period of sample collection can also influence the observed resistance rates. However, these studies, including ours, consistently found that agents from the carbapenem group remain effective.

Notably, 20.93% (9/43) of *E. coli* isolates in this study were MDR and phenotypically identified as ESBL producers. This prevalence is comparable to that reported for *Enterobacteriaceae* from dog fecal samples in Greece (20.40%) [18] but higher than rates observed in other countries, such as 11.8% in healthy dogs in Portugal [19], 11.70% in dogs and cats in Algeria [20], and 11.54% in dogs in the UAE [17]. While the CTX-M type is currently the most prevalent ESBL globally, the TEM type predominated in our study. This finding aligns with a previous study in Thailand [15], which reported the highest rate of *bla*_TEM_ (24.87%) in *E. coli* isolated from dogs, followed by *bla*_CTX-M_ (21.69%) and *bla*_SHV_ (8.99%). Interestingly, for *E. coli* isolated from cats in the same study, *bla*_CTX-M_ was more prevalent, with a rate of 71.97%, followed by *bla*_TEM_ at 43.18%. In contrast, studies conducted in Europe and other Asian countries have consistently identified *bla*_CTX-M_ and its variants as the most prevalent ESBLs in *E. coli* isolated from companion animals [19,21,22,23]. The detection of the *bla*_SHV_ gene in only one *E. coli* isolate in this study suggests a low prevalence among companion animals, aligning with previous studies that report *bla*_SHV_ at lower rates than other ESBL genes like *bla*_CTX-M_ and *bla*_TEM_ [24,25]. However, *bla*_SHV_ prevalence varies by region, species, and antimicrobial use, with 40.5% reported in *E. coli* from companion animals in Shandong, China, and 8.1% in beef cattle from the Sichuan–Chongqing Circle [26,27]. Although *bla*_SHV_ is more commonly associated with *Klebsiella pneumoniae*, its detection in *E. coli* highlights the potential for cross-species transmission, underscoring the need for continued surveillance and further research into its spread mechanisms [28]. This discrepancy highlights the potential for significant variations in ESBL types across different animal species and geographical locations. The presence of ESBL-producing *E. coli* complicates treatment options for infections. Antibiotics such as third-generation cephalosporins, which are commonly used in veterinary medicine, become ineffective, leaving fewer therapeutic choices. Antimicrobial agents classified in carbapenems, which remain effective, are often reserved for human medicine, raising ethical and regulatory challenges in their use for companion animals. Additionally, further research is needed to understand the genetic mechanisms driving ESBL dissemination and to explore alternative treatment options, such as bacteriophages or natural antimicrobial agents, to combat resistant strains. These efforts will be crucial in mitigating the impact of ESBL-producing *E. coli* on both animal and human health.

The exploration of algal extracts as alternative antimicrobial agents offers great promise. In this study, the ethanol extract of *H. pluvialis* (HE) at 1.55 mg/mL and *C. lentillifera* (CE) at 3.10 mg/mL demonstrated notable antibacterial activity against MDR *E. coli* isolates. Supporting this, a previous study reported that high-polarity ethanol extract from red *Haematococcus* exhibited greater antimicrobial activity than a low-polarity hexane extract [29], likely due to a complex composition of short-chain fatty acids, such as propanoic, lactic, and butanoic acids [30]. Additionally, ultrasonic-assisted ethanol extraction from *H. pluvialis* has been shown to effectively release astaxanthin, a reddish pigment categorized among bioactive carotenoids with notable antioxidant properties [31]. Previous studies have also reported that extracts from *H. lacustris* exhibit novel activity against multi-antibiotic-resistant microbes [32]. Furthermore, the antimicrobial efficacy of ethanol extracts from *H. pluvialis* was found to surpass that of hexane extracts [29]. Moreover, astaxanthin derived from *H. lacustris* has demonstrated potential effectiveness when examined with antibiotics such as ampicillin, chloramphenicol, and penicillin [33]. Consistently, previous studies [32,34,35] reported that the N-hexane and methanol extracts of *Haematococcus lacustris* and *C. lentillifera*, displayed moderate to significant inhibitory effects on *E. coli*, further suggesting the potential of algal compounds as alternative treatments. Previous reports of the maceration of *C. lentillifera* using ethanol extract showcased a range of metabolite profiles, which included 3-[3-(beta-d-glucopyranosyloxy)-2-hydroxyphenyl]propanoic acid, choline, betaine, 2-(1*H*-indol-3-yl)-3-[4-(trifluoromethyl)phenyl]acrylonitrile, 2-(3,4 dihydroxyphenyl)acetamide, isoamylamine, palmitoleic acid, and α-linolenic acid. These compounds serve as novel bioactive agents for potential applications in cancer treatment, particularly against hepatoma, breast, colorectal, and even leukemia cancers [36]. Antibacterial compounds derived from Chlorophyta extracts have been shown to be effective against both Gram-positive and Gram-negative bacteria [37]. These findings suggest that algal extracts, particularly from *H. pluvialis* and *C. lentillifera* have the potential to serve as adjuncts or alternatives to traditional antibiotics in combating MDR bacteria. Further research is crucial to isolate and characterize the active antibacterial compounds, assess their clinical safety and efficacy, and determine optimal concentrations for use. Such targeted investigations will be essential for refining these bioactive compounds for applications in both clinical and environmental settings. The growing prevalence of MDR *E. coli* poses a significant challenge, but algal extracts present a promising avenue for the development of innovative antibacterial agents.

## 4. Materials and Methods

### 4.1. Sample Collection and Identification of E. coli

In this study, rectal swab samples were collected from 53 clinically healthy dogs at 10 veterinary hospitals and clinics in Bangkok and its surrounding areas. This study was conducted in accordance with ethical guidelines and regulations for the use of animals in research. The research protocol was reviewed and approved by the Kasetsart University Institutional Animal Care and Use Committee (Approval Number: U1-00002-2558). After collection, the swabs were transported to the laboratory within 24 h and stored at 4 °C until testing. Each swab was streaked onto MacConkey agar (Himedia, Mumbai, India) and incubated at 37 °C for 24 h. Colonies suspected to be *E. coli*, based on positive lactose fermentation, were selected and streaked onto eosin methylene blue agar (Himedia, Mumbai, India). Identification of *E. coli* was confirmed using Gram staining and conventional biochemical tests, including the indole, Methyl Red, Voges–Proskauer, and citrate utilization tests. Additionally, PCR targeting *uidA* was performed for molecular confirmation of *E. coli* isolates using the primers listed in Table 4 [38]. *E. coli* ATCC 25922 was used as a positive control strain. Confirmed *E. coli* isolates were stored in Luria–Bertani broth (Himedia, Mumbai, India) containing 30% glycerol (Sigma-Aldrich, Inc., St. Louis, MO, USA) at −80 °C (Thermo Scientific, Waltham, MA, USA) for further analysis.

### 4.2. Antimicrobial Susceptibility Testing

The antimicrobial susceptibility profiles of the isolated *E. coli* strains were determined using the disk diffusion method, and the results were interpreted according to the Clinical and Laboratory Standards Institute (CLSI) M100 guidelines [39]. Briefly, *E. coli* isolates were adjusted to a concentration of 0.5 McFarland standard and streaked onto Mueller–Hinton agar (MHA) (Oxoid, Ogdensburg, NY, USA). Eleven antimicrobial agents (Oxoid, USA) from eight drug classes were tested: penicillin (ampicillin, AMP 10 µg); β-lactam combination agents (amoxicillin–clavulanate, AMC 20/10 µg); cephems (cefepime, FEP 30 µg; cefotaxime, CTX 30 µg; ceftriaxone, CRO 30 µg); penems (meropenem, MEM 10 µg); aminoglycosides (gentamicin, CN 10 µg; amikacin, AK 30 µg); tetracyclines (tetracycline, TE 30 µg); fluoroquinolones (ciprofloxacin, CIP 5 µg); and folate pathway antagonists (trimethoprim–sulfamethoxazole, SXT 1.25/23.75 µg). Antimicrobial disks were placed on the agar surface, and the plates were incubated at 37 °C for 24 h. *E. coli* ATCC 25922 was used as a quality control strain. The diameters of the zones of inhibition around each disk were measured and interpreted as susceptible (S), intermediate (I), or resistant (R) according to established criteria. Isolates resistant to at least three different antimicrobial classes were classified as MDR [40].

### 4.3. Multiple Antibiotic Resistance (MAR) Index Calculation

In a previous study [16], the MAR index was calculated using the formula MAR = a/b, where a represents the number of antibiotics to which an isolate was resistant, and b represents the total number of antibiotics tested.

### 4.4. Detection of ESBL-Producing E. coli

ESBL-producing *E. coli* isolates were phenotypically screened using the double-disk synergy test with cefotaxime (CTX 30 µg) and cefotaxime–clavulanic acid (CTX/CV 30/10 µg), as well as ceftazidime (CAZ 30 µg) and ceftazidime–clavulanic acid (CAZ/CV 30/10 µg) (Mast Group, Bootle, UK), following CLSI guidelines [39]. Briefly, *E. coli* isolates were adjusted to a concentration of 0.5 McFarland standard and streaked onto MHA. The antimicrobial agent disks were placed on the agar surface and incubated at 37 °C for 24 h. *E. coli* ATCC 25922 was used as a quality control strain. A difference of ≥5 mm in zone diameter between the agent disk tested in combination with clavulanate and the agent disk tested alone was considered indicative of ESBL production. Additionally, CHROMID^®^ ESBL agar (bioMérieux, Marcy l’Étoile, France) was used to confirm ESBL expression.

### 4.5. Detection of Beta-Lactamase Genes from MDR E. coli Isolates

DNA extraction for PCR assays was carried out using InstaGene™ Matrix (Bio-Rad, Hercules, CA, USA), following the manufacturer’s instructions. The extracted DNA was stored at −20 °C until use.

The detection of β-lactamase genes (*bla*_TEM_, *bla*_CTX-M_, and *bla*_SHV_) in *E. coli* isolates was performed using multiplex PCR with the primers listed in Table 4. The multiplex PCR conditions adapted from [41] and were as follows: each reaction mixture had a final volume of 15 µL, consisting of Thermo Scientific^®^ DreamTaq Green PCR Master Mix (1× final concentration; Thermo Scientific, Waltham, MA, USA), 0.2 µM of each primer (*bla*_TEM_, *bla*_CTX-M_, and *bla*_SHV_), and 2 µL of the DNA sample. PCR reactions were performed in a thermal cycler (SensoQuest, Göttingen, Germany) under the following conditions: initialization at 95 °C for 3 min, followed by 30 cycles of denaturation at 95 °C for 30 s, annealing at 55 °C for 30 s, and extension at 72 °C for 1 min. After the cycles, a final extension was performed at 72 °C for 7 min, and the PCR products were stored at −20 °C. *E. coli* ATCC 25922 was used as a negative control, while previously confirmed positive isolates served as positive controls for PCR validation in this study [42]. The PCR amplicons were electrophoretically separated using a 1.5% (*w*/*v*) agarose gel stained with SYBR Safe (Thermo Scientific, Waltham, MA, USA).

**Table 4:** List of primers used in this study to detect beta-lactamase genes.

Genes	Primer Sequences (5′-3′)	Estimated Product Size (bp)	Reference
*uidA*	F-TGGTAATTACCGACGAAAACGGC	147	[38]
	R-ACGCGTGGTTACAGTCTTGCG		
*bla*_TEM_	F-ACGCTCACCGGCTCCAGATTAT	445	[41]
	R-TCGCCGCATACACTATTCTCAGA		
*bla*_CTX-M_	F-ATGTGAGYACCAGTAARGTGAT	593	[43]
	R-TGGGTRAARTARGTSACCAGAAT		
*bla*_SHV_	F-TGCTTTGTTATTCGGGCCAA	747	[44]
	R-ATGCGTTATATTCGCTGTG		

### 4.6. Preparation of Crude Extracts

#### 4.6.1. Biomass Preparation

Five distinct strains of cyanobacteria, microalgae, and green algae were utilized for the extraction of bioactive substances. The cyanobacteria strain used was *Arthrospira platensis* IFRPD 1182. The microalgae strains included *Haematococcus pluvialis* and *Nannochloropsis oculata*, while the green algae strains were *Ulva rigida* and *Caulerpa lentillifera*. The *A. platensis* IFRPD 1182 was maintained by the Institute of Food Research and Product Development at Kasetsart University in Thailand. *A. platensis* was cultured in Zarrouk medium and incubated in chamber equipment under previously reported conditions, which were expanded to allow for biomass production in a 200 L raceway pond [45]. The biomass from *A. platensis* was collected and dehydrated using a hot air oven at 65 °C for 6 h. It was then milled to achieve a uniform size of 0.5 mm using a mill grinder. Additionally, other microalgae, such as *H. pluvialis*, a red microalga rich in astaxanthin, and *N. oculata*, a green microalga, were purchased by T.S. Twin Product Company Limited in Thailand.

The macroalgae Sea Lettuce *U. rigida* was obtained from the Phetchaburi Coastal Aquacultural Research and Development Center, Department of Fisheries, in Phetchaburi, Thailand. The culture conditions were controlled as stated in the previous report [46]. Premium food-grade Sea Grapes, *C. lentillifera*, were purchased from an organic algae farm in Phetchaburi, Thailand [47]. Both macroalgae were dried using a hot air oven at 65 °C for 6 h and then milled to achieve an equal size of 0.5 mm using a mill grinder.

#### 4.6.2. Extraction Procedures

The crude extraction process was conducted in two stages. In the first stage, biomass was extracted using 70% ethanol at a 1:50 (*w*/*v*) ratio in an ultrasonic bath (DT 100 H, Berlin, Germany) set at 35 kHz and 320 W for 20 min. The ethanolic extract was then separated by centrifugation (Frontier™ 2000 Multi-Centrifuge, Ohaus, NJ, USA) at 3660× *g* for 20 min. The resulting supernatant was collected and evaporated using a rotary evaporator (Rotavapor^®^ R-300, Flawil, Switzerland). The second stage involved enzymatic hydrolysis of the remaining cell residues. These residues were resuspended in 0.01 M phosphate buffer (pH 7.0) and treated with 3% (*w*/*v*) Alcalase^®^ enzyme (Novozyme, Aesch, Switzerland). The mixture was incubated at 50 °C for 6 h, followed by enzyme denaturation at 95 °C for 10 min. The hydrolysate was then separated by centrifugation (Frontier™ 2000 Multi-Centrifuge, Ohaus, NJ, USA) at 3660× *g* for 20 min. The supernatant was collected and freeze-dried (VFD-12SH; Grisrianthon Co., Bangkok, Thailand) at 30–60 Pa for 24 h. The freeze-dried sample was subsequently milled to a uniform particle size of 0.5 mm. Finally, the ethanolic extract and freeze-dried protein hydrolysate from each sample were dissolved in 25% DMSO, protected from light using aluminum foil, and stored at −20 °C for future experiments. Table 5 provides details of the algae extraction samples.

### 4.7. Antibacterial Activity of Algal Extracts Against Multidrug-Resistant E. coli Isolates

The antibacterial activity of algal extracts was evaluated against both multidrug-resistant and ESBL-producing *E. coli* isolates using the broth microdilution method in a sterile 96-well microtiter plate, following the standard protocols, with modifications where necessary [48]. Each well was initially filled with 50 µL of cation-adjusted Mueller–Hinton Broth (CAMHB). Subsequently, 50 µL of the algal extracts and controls were added to the wells as follows: SE to row 1, CE to row 2, NE to row 3, HE to row 4, UE to row 5, FS to row 6, FC to row 7, FN to row 8, FH to row 9, FU to row 10, 25% DMSO was set as the negative control at row 11, and CAMHB was set as the positive control to row 12.

*E. coli* colonies were suspended in normal saline to match the turbidity of a 0.5 McFarland standard, then further diluted 1:20 in CAMHB. Subsequently, 10 µL of the *E. coli* suspension was added to each well to achieve a final concentration of approximately 5 × 10^5^ CFU/mL per well. The final concentration of each algal extract was determined based on the preliminary results with *E. coli* ATCC 25922. The plates were covered and incubated at 37 °C (Memmert, Büchenbach, Germany) for 24 h.

The dark pigmentation of the algal extracts obscured direct observation of inhibition in the 96-well plate. Therefore, 20 µL from each well was streaked in a straight line onto MHA and incubated overnight at 37 °C to assess bacterial growth. All experiments were performed in triplicate to ensure reliability.

## 5. Conclusions

Our findings demonstrate a high prevalence of MDR *E. coli* in healthy dogs in Thailand, highlighting the urgent need to address antimicrobial resistance in companion animals. Among the isolates, 67.44% were identified as MDR, exhibiting resistance to a wide range of antibiotics, with the highest resistance observed against ampicillin (79.07%) and tetracycline (65.12%), while the lowest resistance was against meropenem (2.33%). Additionally, 31.03% of isolates were found to produce ESBL, with a notable presence of *bla*_TEM_ (53.49%) and *bla*_CTX-M_ (9.3%) genes, indicating the potential for gene transfer of resistance traits. This finding is particularly concerning, as ESBL production significantly limits treatment options and increases the risk of zoonotic transmission. The assessment of algal extracts revealed that ethanol extracts of *H. pluvialis* and *C. lentillifera* exhibited inhibitory effects against MDR *E. coli* isolates, suggesting the potential of these algae as sources of novel antibacterial agents. However, the extracts of *A. platensis* showed limited inhibition, while *U. rigida* and enzymatic hydrolysis of *A. platensis*, *N. oculata*, and *U. rigida* did not demonstrate an inhibitory effect. These findings highlight the potential of certain algal extracts as promising antimicrobial agents against MDR *E. coli*. However, further investigation, including in vivo studies, is needed to optimize their efficacy and explore their potential for clinical and environmental applications.

## Figures and Tables

**Table 1:** Antimicrobial susceptibility profiles of *E. coli* isolates to various antimicrobial agents.

Antimicrobial Class	Antimicrobial Agent (µg)	No. of Resistant Strains (%)	No. of Intermediate Strains (%)	No. of Susceptible Strains (%)
Penicillin	Ampicillin (10)	34 (79.07)	0 (0)	9 (20.93)
β-Lactam combination agents	Amoxicillin–clavulanate (20/10)	16 (37.21)	4 (9.3)	23 (53.49)
Cephems	Cefepime (30)	8 (18.60)	4 (9.3)	31 (72.09)
	Cefotaxime (30)	14 (32.56)	2 (4.65)	27 (62.79)
	Ceftriaxone (30)	14 (32.56)	1 (2.33)	28 (65.12)
Carbapenem	Meropenem (10)	1 (2.33)	7 (16.28)	35 (81.40)
Aminoglycosides	Gentamicin (10)	16 (37.21)	1 (2.33)	26 (60.47)
	Amikacin (30)	7 (16.28)	5 (11.63)	31 (72.09)
Tetracycline	Tetracycline (30)	28 (65.12)	2 (4.65)	13 (30.23)
Fluoroquinolones	Ciprofloxacin (5)	27 (62.79)	9 (20.93)	7 (16.28)
Folate pathway antagonist	Trimethoprim–sulfamethoxazole (1.25/23.75)	26 (60.47)	2 (4.65)	15 (34.88)

**Table 2:** Antibiotic resistance patterns, multiple antibiotic resistance (MAR) index, and beta-lactamase gene profiles of the *E. coli* isolates.

Isolate	Antibiotic Agents											MAR Index	Genes
	CRO	CTX	FEP	AMC	AMP	TE	MEM	CIP	CN	AK	SXT		
En1	S	S	S	R	R	S	S	R	S	S	R	0.36	*bla*_TEM_
En2	R	R	R	R	R	R	I	R	R	S	R	0.82	*bla*_TEM_
En3 **	R	R	R	R	R	R	I	R	R	S	S	0.73	*bla*_TEM_
En6 *	S	S	S	S	S	S	S	S	R	S	S	0.09	ND
En7	R	R	S	R	R	R	S	R	S	R	I	0.64	ND
En8 **	R	R	R	R	R	R	S	R	R	S	R	0.82	*bla*_CTX-M_
En9 *	S	S	S	S	S	S	S	R	S	S	S	0.09	ND
En12	S	S	S	S	R	R	S	R	S	R	R	0.45	*bla*_TEM_
En13	S	S	S	R	R	R	S	R	S	S	S	0.36	*bla*_TEM_
En14 **	R	R	I	R	R	R	S	R	R	S	R	0.73	*bla*_TEM_
En15	I	R	I	R	R	R	S	R	R	S	R	0.64	*bla*_TEM_
En16 **	R	R	S	R	R	R	I	R	R	R	R	0.82	*bla*_TEM_
En17	R	R	R	R	R	R	I	R	R	R	R	0.91	ND
En19 *	S	S	S	S	S	S	S	I	S	S	S	0.00	ND
En20 *	S	S	S	I	R	S	S	I	S	S	R	0.18	*bla*_TEM_
En21	S	S	S	I	R	R	S	I	S	S	R	0.27	*bla*_TEM_
En22 *	S	S	S	S	S	S	S	S	S	R	S	0.09	ND
En23 *	S	S	S	S	R	R	I	S	S	I	S	0.18	*bla*_TEM_
En24 **	R	R	R	R	R	R	I	R	R	S	R	0.82	*bla*_TEM_
En25	S	S	S	S	R	R	S	R	S	S	R	0.36	*bla*_TEM_
En26	S	S	S	I	R	R	S	R	R	S	R	0.45	*bla*_TEM_
En27 *	S	S	S	S	S	S	S	I	S	S	I	0.00	ND
En28 *	S	S	S	S	R	I	S	I	S	S	R	0.18	*bla*_TEM_
En29 **	R	I	S	R	R	R	R	R	I	I	S	0.55	*bla*_CTX-M_
En30	S	S	S	S	R	S	S	S	R	S	R	0.27	*bla*_TEM_
En32	S	I	I	R	R	R	S	R	R	S	R	0.55	*bla*_CTX-M_
En33	R	R	R	R	R	R	I	R	R	I	R	0.82	ND
En34 **	R	R	I	R	R	R	S	R	S	S	R	0.64	*bla*_TEM_
En35	S	S	S	S	S	R	S	R	S	S	R	0.27	ND
En36 **	R	R	R	S	R	R	S	R	R	S	S	0.64	*bla*_CTX-M_
En37 *	S	S	S	S	S	R	S	S	S	S	S	0.09	*bla*_TEM_
En38	S	S	S	S	R	R	S	S	S	S	R	0.27	*bla*_TEM_
En41 *	S	S	S	S	S	S	S	S	S	S	S	0.00	*bla*_TEM_
En42 *	S	S	S	S	R	S	S	I	S	S	S	0.09	ND
En43 *	S	S	S	S	R	S	S	I	S	I	S	0.09	*bla*_SHV_
En45	R	R	S	R	R	R	S	R	R	R	R	0.73	*bla*_TEM_
En46 **	R	R	R	I	R	I	S	R	R	S	R	0.64	ND
En47	S	S	S	S	R	R	S	R	S	S	R	0.36	*bla*_TEM_
En48	S	S	S	S	R	R	S	R	S	S	R	0.36	*bla*_TEM_
En49	S	S	S	S	R	R	S	R	S	S	R	0.36	ND
En50 *	S	S	S	S	R	S	S	I	S	S	S	0.09	ND
En51 *	S	S	S	S	S	S	S	I	S	I	S	0.00	ND
En52	S	S	S	S	R	R	S	R	S	R	R	0.45	ND

* Indicates non-MDR *E. coli* isolates; ** indicates ESBL-producing *E. coli* isolates. Abbreviations: CRO, ceftriaxone; CTX, cefotaxime; FEP, cefepime; AMC, amoxicillin–clavulanate; AMP, ampicillin; TE, tetracycline; MEM, meropenem; CIP, ciprofloxacin; CN, gentamicin; AK, amikacin; SXT, trimethoprim–sulfamethoxazole; S: susceptible; I: intermediate; R: resistant; ND: not detected.

**Table 3:** The effect of algal extracts against multidrug-resistant *E. coli* isolates.

Algal Extracts	Concentration in 12.5% DMSO (mg/mL)	Number of Multidrug-Resistant *E. coli* Isolates Affected by Algal Extracts		
		Inhibiting	Slightly Inhibiting	Non-Inhibiting
SE	1.43	0	4 (13.79%)	25 (86.21%)
HE	1.55	3 (10.34%)	9 (31.03%)	17 (58.62%)
NE	2.94	0	1 (3.45%)	28 (96.55%)
UE	3.21	0	0	29 (100%)
CE	3.10	1 (3.45%)	0	28 (96.55%)
FS	3.28	0	0	29 (100%)
FH	2.75	0	4 (13.79%)	25 (86.21%)
FN	1.65	0	0	29 (100%)
FU	2.28	0	0	29 (100%)
FC	1.40	0	1 (3.45%)	28 (96.55%)

SE, HE, NE, UE, and CE indicate the ethanol extraction of *Arthrospira platensis*, *Haematococcus pluvialis*, *Nannochloropsis oculata*, *Ulva rigida*, and *Caulerpa lentillifera,* respectively, then FS, FH, FN, FU, and FC indicate the enzymatic hydrolysis of *Arthrospira platensis*, *Haematococcus pluvialis*, *Nannochloropsis oculata*, *Ulva rigida*, and *Caulerpa lentillifera*, respectively.

**Table 5:** Crude extracts from various strains of algae.

Algae	Abbreviation of	
	Ethanol Extraction	Enzymatic Hydrolysis
*Arthrospira platensis*	SE	FS
*Haematococcus pluvialis*	HE	FH
*Nannochloropsis oculata*	NE	FN
*Ulva rigida*	UE	FU
*Caulerpa lentillifera*	CE	FC

## Data Availability

The authors confirm that the data supporting the findings of this study are available within the article.
